# Prevalence and risk factors for vitamin D insufficiency and deficiency at birth and associated outcome

**DOI:** 10.1186/s12887-016-0741-4

**Published:** 2016-12-08

**Authors:** Ian Marshall, Rajeev Mehta, Charletta Ayers, Smita Dhumal, Anna Petrova

**Affiliations:** Department of Pediatrics, Rutgers - Robert Wood Jonson Medical School, 89 French Street, New Brunswick, NJ 08901 USA

**Keywords:** Cord blood, Vitamin D, Deficiency, Insufficiency

## Abstract

**Background:**

Occurrence and consequence of cord blood (CB) vitamin D insufficiency/deficiency has not been adequately explored despite rising concern regarding this topic in pediatrics. This study was designed to determine the rate, maternal risk factors, and clinical outcomes in infants in association with vitamin D insufficient/deficient status at birth.

**Methods:**

American Academy of Pediatrics (AAP) defined levels (ng/mL) were utilized to categorize the vitamin D status in CB samples as deficient (5–15), insufficient (16–20), and sufficient (21–100). We used descriptive statistics and multiple regression models to identify the rate and factors associated with vitamin D deficiency/insufficiency and related outcomes in the enrolled mother-infant pairs.

**Results:**

This prospective study was conducted at a single center on postpartum women and their infants. Vitamin D deficiency and insufficiency was recorded in 38.9 and 29.8% respectively of the 265 CB samples. Deficient CB vitamin D levels in infants were associated with maternal Black, Hispanic, or Asian race/ethnicity, younger age, and increased number of pregnancies. The likelihood for infants to be born with an insufficient vitamin D level increases with younger maternal age and the number of pregnancies as well as Asian ethnicity. We did not find an association between the vitamin D status at birth and pre-discharge clinical characteristics of the neonates.

**Conclusions:**

The likelihood for an infant to be born with vitamin D deficiency/insufficiency is relatively high and is related mainly to younger maternal age, gravidity, and non-White race/ethnicity. Our findings raise a question regarding the adequacy of the AAP recommended vitamin D supplementation requirements without knowing the infant’s vitamin D status at birth.

## Background

Characterization of the vitamin D status in the maternal-fetal-neonatal interface is clinically important because of the increased rates of detection of vitamin D insufficiency in cord blood (CB) and the associated risk for health complications in infants and children [1, 2], including but not limited to rickets [3], infectious and allergic diseases, and type 1 diabetes [4–6]. Assessing vitamin D status using CB is a challenge as the optimal level of vitamin D in children is still the subject of discussion [7]. For the child population, t he American Academy of Pediatrics (AAP) defines a vitamin D level of 20 ng/mL as sufficient [8]. The Drug and Therapeutics Committee of the Lawson Wilkins Pediatric Endocrine Society classified vitamin D levels < 5 ng/mL in children as severely deficient, 5–15 ng/mL as deficient, and 15–20 ng/mL as insufficient [9]. However, the Clinical Practice Guidelines published by the Endocrine Society recommends that 30 ng/mL of vitamin D in children and adults should be considered sufficient and classifies levels below 20 ng/mL (50 nmol/L) and 21–29 ng/mL (55–75 nmol/L) as deficient and insufficient, respectively [10].

Several studies were designed to identify the rates of vitamin D insufficiency in CB of infants born to different maternal populations. A CB vitamin D level ≤ 20 ng/mL was detected in 84% of African American and 22% of White infants born in Florida [11], 55% of infants from the mixed ethnic population in Belgium [12], and 55.5% of infants born to Italian women and 77.3% of the immigrant mothers in Italy from Latin, Asian and African countries [13]. Bodnal et al. [14] reported CB vitamin D deficiency (<15 ng/mL) and insufficiency (15–32 ng/mL) in 45.6 and 46.8% of African American infants, and 9.7 and 56.4% of White neonates from the northern United States. Vitamin D deficiency [less than 25 nmol/L (<10 ng/mL)] and insufficiency [between 25 and 50 nmol/L (10–20 ng/mL)] have been reported in CB samples of 25 and 44% infants born to European, and 75 and 21% of infants born to non-European mothers in Netherlands [15]. A population-based study from Australia reported vitamin D levels of ≤ 10 ng/mL (≤25 nmol/L) in 11% of the cord blood samples [16]. CB samples from multiethnic infants living in the tropical region showed deficient vitamin D levels (<20 ng/mL) in 28% and insufficient levels (20–30 ng/mL) in 50% of the cases [17].

Differences in the classification [8–10] used to assess the infants’ vitamin D status and strong dependence of their vitamin D pool on maternal vitamin D levels [18] may be responsible for the wide variability in the reported rates of vitamin D deficiency/insufficiency in infants at birth. Despite numerous publications that explored vitamin D status in CB, further research is required to determine the likelihood for detection of vitamin D insufficiency in CB of infants. Improved understanding of factors associated with development of vitamin D deficiency/ insufficiency will facilitate the creation and implementation of appropriate preventive strategies.

To the best of our knowledge, our study is the first that in addition to identifying the rate, also estimates the risk of vitamin D deficiency/insufficiency in the CB and associated outcome in a diverse population of infants.

## Methods

This prospective cohort study was conducted at a single center on a non-random sample of postpartum women who were aged more than 18 years old and delivered healthy infants at Robert Wood Johnson University Hospital between July 2014 and June 2015. The study was approved by the Rutgers Robert Wood Johnson Medical School’s Institutional Review Board. Signed informed consent by the mother was required for participation in this study.

### Clinical data collection

We used maternal and neonatal medical records to extract significant demographic and medical information. A standardized data extraction tool was used to collect relevant maternal (age, weight at delivery, race/ethnicity, insurance status, use of prenatal vitamins and/or other medications, evidence of pregnancy complications, mode of delivery) and neonatal (gestational age, birth weight and length, Apgar score, morbidity during birth hospitalization) demographic and clinical information.

### Samples proceed and testing procedure

Leftover samples in lavender top EDTA (ethylenediaminetetraacetic acid) tubes from routine collection of CB for identification of blood type in all infants born in the hospital were used to test for vitamin D levels. The samples were centrifuged to derive plasma that was divided into three aliquots and stored in deep freezer at −70°∁ pending simultaneous testing in a reference laboratory.

The reference laboratory used Advanced Liquid Chromatography/Tandem Mass Spectrometry (LC/MS/MS) to measure total 25-hydroxyvitamin D [25(OH)D] along with vitamin D_3_ and D_2_. This methodology differs from standard LC/MS/MS assays because it enables chromatographic separation of the C3-epimer from 25-OH Vitamin D, which is the low activity form of the potentially interfering [25(OH) D_3_] metabolite [19]. It has been shown that the C3-epimer of [25(OH) D_3_] accounts for up to 14 and 25% of total levels of [25(OH) D_3_] in cord and infant blood, respectively [20]. Hence, elimination of the low activity form of [25(OH) D_3_] from the final report prevents false elevation of results. Tested levels of calcidiol [25(OH)D] and vitamin D components, including the ergocalciferol (Vitamin D_2_) and cholecalciferol (Vitamin D_3_) were expressed in ng/mL. The lowest detectable concentrations were 4 ng/mL for both Vitamin D_2_ and D_3_. Vitamin D_2_ levels were undetectable (<4 ng/mL) in 99.9% of CB samples. Therefore, the total level of CB vitamin D measured in this study is equal to [25(OH)D_3_] levels, which is the main contributor to the [25(OH)D] pool.

### Data presentation and statistical analyses

The results of [25(OH) D] were analyzed as a continuous variable and were also stratified with respect to the AAP’s definition of vitamin D status [8, 9] as severe (<5 ng/mL) or mild/moderate deficient (5–15 ng/mL), insufficient (16–20 ng/mL), and sufficient (21–100 ng/mL). Because a severely deficient CB Vitamin D level (<5 ng/mL) was found in only one sample, it was merged with the group that showed deficient [25(OH) D] levels. We performed analysis of variance (ANOVA) and Chi-square test statistics to compare continuous and categorical data between the groups with respect to the infants’ vitamin D status at birth defined as deficient, insufficient, and sufficient. Variables with significant between-group variability (*P* value of less than 0.05) were classified for inclusion into the multiple regression models to estimate their independent role in the development of vitamin D deficiency and insufficiency in CB as compared to those with sufficient levels of vitamin D. In addition multiple regression models were stratified by race/ethnicity defined as White, Black, Hispanic, and Asian. Mothers without information on race/ethnicity were excluded from subgroup analysis. Possible multicollinearity of the predictive value of the variables included in the regression model was assessed by estimation of variance inflation factor (VIF/(1-*R* ^*2*^)], where *R* ^*2*^ is coefficient of determination, and VIF above 2.5 was classed as indicative of a possible linear dependence of the regression coefficient with other predictors [21].

Data are presented as means, proportion (%), regression coefficient (β) ± standard error (Std. Err. of β), and Odds Ratio (OR) and 95% Confidence intervals (95% CI). Difference was considered significant if *P* value was less than 0.05. STATISTICA version 12.0 (StatSoft Inc, Tulsa, OK) was used to analyze the data.

## Results

Among the 360 enrolled mothers, a total of 265 mother-infant pairs were included in the final analysis. The difference is due to technical problems with collection of 92 leftover CB samples, and insufficient quantity of the specimen for testing of vitamin D levels in three CB samples. Complete demographic and clinical data collection was available for 265 mother-infant pairs. In 21 subjects (7.9%), maternal race/ethnicity information was not verifiable.

### Characteristics of umbilical cord blood vitamin D status

**Fig. 1:**
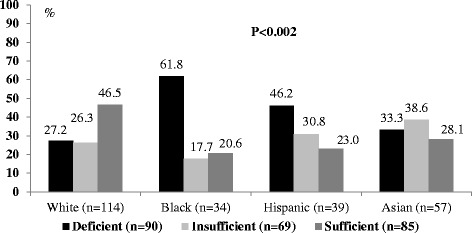
Cord blood 25(OH)D status and maternal race/ethnicity. Chi-square test statistics were used to compare CB vitamin status defined as deficient, insufficient and sufficient with respect to maternal race/ethnicity

CB levels of vitamin D were classified as deficient, insufficient, and sufficient in 95 (38.9%, 95%CI 33.0–44.8), 79 (29.8%, 95%CI 24.3–35.3), and 91 (34.3%, 95%CI 28.6–40) infants, respectively. The mean and 95% CI of mean of CB vitamin D levels in infants defined with sufficient, insufficient, and deficient levels was 27.3 ng/mL (26–28.5), 17.8 ng/mL (17.4–18.1), and 11.6 ng/mL (11–12.2), respectively. As shown in Table 1, mothers of infants with deficient and insufficient level of vitamin D in CB were younger and had a greater number of pregnancies as compared to mothers of infants with sufficient level of [25(OH)D]. In addition, we identified significant variability in the infant’s vitamin D level at birth due to maternal race/ethnicity (Fig. 1). The highest prevalence of vitamin D deficiency was recorded in CB of infants born to Black mothers (61.8%) and vitamin D sufficiency in infants born to White mothers (46.5%).

**Table 1:** Maternal demographics and clinical characteristics with respect to CB vitamin D status

Parametric	Vitamin D status			*P* value
	Deficiency *N* = 95	Insufficiency *N* = 79	Sufficiency *N* = 91	
Age (years)	30.6+/−5.7 (29.4–31.8)	29.9+/−5.1 (28.8–31.1)	32.6+/−5.3 (31.5–33.8)	<0.01
Medicaid/uninsured	16.0% (9.9–24.7)	8.8% (4.3–16.9)	6.7% (3.1–13.6)	0.09
Weight at birth (pounds)	175+/−41 (167–183)	172+/−34 (165–180)	174+/−39 (166–182)	0.89
Multiple gestation	1.1% (0.002–0.06)	1.3% (0.002–0.07)	3.3% (0.11–0.09)	0.47
Nulliparity (%)	27.7% (19.6–37.4)	38.8% (28.8–49.7)	36.3% (27.1–46.5)	0.26
First gravidity (%)	21.3% (9.1–23.5)	26.3% (14.7–32.8)	31.9% (6.9–20.4)	0.56
Gravidity (number)	3.4+/−2.4 (2.9–3.9)	2.8+/−1.9 (2.3–3.2)	2.4+/−1.3 (2.1–2.6)	<0.01
Gestational age	38.9+/−1.3 (38.6–39.1)	39.2+/−1.2 (38.9–39.5)	39.0+/−1.3 (38.7–39.3)	0.15
Winter season deliveries	14.9% (9.1–23.5)	22.5% (14.7–32.8)	12.1% (6.9–20.4)	0.17
Cesarean section	32.9% (24.3–42.9)	35.0% (25.5–45.9)	41.8% (32.2–52.1)	0.44
Vitamins supplementation	66.0% (60.3–78.5)	77.5% (67.2–85.3)	67.0% (56.9–75.8)	0.20
Iron supplementation	8.5% (4.4–15.9)	3.8% (1.3–10.5)	2.2% (0.6–7.7)	0.15
Thyroid medications	9.6% (5.1–17.2)	8.8% (4.3–16.9)	8.8% (4.5–16.4)	0.98
Substance abuse (smoking/alcohol/drugs)	3.2% (1.1–8.9)	5.0% (2.0–12.2)	6.6% (3.1–13.6)	0.56
Morbidities during pregnancy	21.3% (14.2–30.6)	26.3% (17.9–36.8)	23.1% (15.6–32.7)	0.74
Diabetes	10.6% (5.9–18.5)	12.5% (6.9–21.5)	7.7% (3.8–15.1)	0.57

Data presented as mean or proportion (%) with 95% confidence interval

### Factors associated with CB vitamin D status

Maternal race/ethnicity, age and gravidity were included in the multiple regression models to identify factors associated with detection of vitamin D deficiency (Model 1) and insufficiency (Model 2) in infants’ CB. In these models, the VIF indicates no multicollinearity between the defined co-variances: 1.08 and 1.03 for race/ethnicity (White vs. non-White), 1.03 and 1.06 for age, 1.07 and 1.01 for gravidity. Inclusion of maternal race/ethnicity (White vs. non-White), age, and gravidity in the multiple regression models revealed independent association of vitamin D deficiency and insufficiency in the infant’s CB with non-White maternal race/ethnicity, younger age, and increased number of pregnancies (Table 1). When the same variables were included in each race/ethnic group (Table 2), the risk for detection of vitamin D deficiency in CB of infants was associated with Black, Asian and Hispanic race/ethnicity of the mothers. Risk for detection of vitamin D insufficiency in CB of infants born to Black or Hispanic mothers was comparable to those in White neonates but higher in babies born to Asian mothers. A comparable magnitude of the independent effect of maternal age and gravidity on vitamin D status was identified in each race/ethnicity model (data not presented).

**Table 2:** Contribution of maternal characteristics in development of CB vitamin D deficiency and insufficiency

Variables	CB vitamin D status			
	Insufficiency		Deficiency	
	β +/−Std. Err	*P*-value	β+/−Std. Err	*P*-value
White (1) / non-White (0)	−0.204 +/− 0.076	0.01	−0.290+/−0.067	0.001
Age (years)	−0.297+/− 0.076	0.001	−0.256 +/0.069	0.0001
Gravidity (number)	0.197 +/− 0.077	0.02	0.346 +/− 0.069	0.0001

Results from multiple regression analysis represented as regression coefficient (β) and Standard Error of better (Std. Err). CB vitamin D status: deficiency (*n* = 95), insufficiency (*n* = 79), sufficient (*n* = 91)

### Neonatal characteristics and CB vitamin D status

Among the 265 infants in this study, 6.4% were born at gestational age between 37 and 34 weeks and 1.9% from multiple gestations. Birth weight of the infants was not less than 2030 g. In 3.8% of infants, the Apgar score at 1 min was less than 7; and 6.8% of the infants were admitted to the neonatal intensive care unit. We found no significant difference in the clinical characteristics of the infants with regards to their CB vitamin D level (Table 3).

**Table 3:** Characteristics of neonates during birth hospitalization with respect to their CB vitamin D status

Parametric	Vitamin D satus			*P* value
	Deficiency *N* = 95	Insufficiency *N* = 79	Sufficiency *N* = 91	
Gestational age (weeks)	38.8 (38.6–39.1)	39.2 (38.9–39.5)	38.9 (38.7–39.3)	0.65
Birth Weight (g)	3301 (3215–3387)	3433 (3322–3544)	3306 (3305–3406)	0.35
Birth length (cm)	48.8 (48.1–49.6)	49.5 (49.0–50.0)	48.3 (47.2–49.5)	0.53
Apgar 1 min <7	2.1% (0.6–7.0)	2.6% (0.7–8.9)	6.5% (2.4–12.2)	0.21
NICU admission	6.3% (0.87–11.7)	5.1% (0–10.6)	8.9% (2.4–15.3)	0.62
Hypoglycemia (%)	0	1.3% (0–4.4)	1.1% (0–3.6)	0.56
Neonatal Jaundice (%)	21.1% (12.6–29.6)	13.9% (5.3–22.5)	13.9% (6.4–21.4)	0.27
Phototherapy (%)	5.3% (0.3–10.3)	1.3% (0–4.4)	1.1% (0–3.6)	0.13

Data presented as mean or proportion (%) with 95% confidence intervals of mean or %

## Discussion

We found that up to 45% infants are at risk for deficient and 35% for insufficient levels of vitamin D. The approximated probability of an infant being born with a sufficient vitamin D level as defined by the AAP for children [8] is less than 40%. Importantly, the upper limit of vitamin D that is defined as sufficient [8] could be suboptimal because vitamin D levels of <30 ng/mL have been associated with increased secretion of intact parathyroid hormone and reduced intestinal calcium absorption in healthy adults [22, 23]. While a vitamin D level of 20 ng/mL is considered sufficient, maintenance of 30 ng/mL is recommended for better health in children and adults [24] despite the lack of clear evidence of an optimal concentration of vitamin D for bone and general health. Because the main source of vitamin D is vitamin D_3_ that is synthesized in the skin through a photolytic reaction, the level of vitamin D depends on skin pigmentation, use of sunscreens, coverage of sun-exposed skin, seasonal factors, and geographic latitude [25–27].

Our findings confirmed previous reports showing an increased risk for vitamin D deficiency in infants born to mothers of non-White race/ethnicity [11, 14, 15, 28]. A study that linked the mean levels of [25(OH)D] in 100 cord plasma samples with maternal ethnicity reported higher level in Caucasians as compared to Asians, Hispanics, Pacific Islanders, and African Americans [17]. To the best of our knowledge, no US study has compared the CB vitamin D levels of infants with respect to the maternal race/ethnicity. Moreover, the internal validity of the existing studies is a subject for discussion because the independent effect of maternal race/ethnicity on the vitamin D status was not controlled for and other maternal factors may also have an association with the development of vitamin D deficiency/insufficiency in infants at birth. We found that independently from the race/ethnicity, younger maternal age and increased number of pregnancies were associated with vitamin D deficiency and insufficiency in CB of the studied infants. Cadario et al. [13] reported an association between younger maternal age and diagnosis of vitamin D deficiency. European data showed both, decreased risk and no effect of parity on vitamin D insufficiency/deficiency in mothers during pregnancy [29, 30]. Unadjusted analysis of the association between clinical data and vitamin D levels in the CB that was conducted on 241 mother-infant pairs (72.6% black and 81.3% multiparous) revealed lower vitamin D levels in the cord blood of nulliparous women [31]. Comparable to previous reports we found no difference between the CB vitamin D status and maternal multivitamin supplementation [30, 32]. However, others have reported that the use of multivitamins, which usually include 400 IU vitamin D_3_, is associated with increased concentrations of 25(OH)D in CB [4]. As in the study by Bodnar et al. [14], we did not find an association between the CB vitamin D level and season of birth.

In the present study, evaluation of the neonatal clinical data did not reveal any obvious association between CB vitamin D levels and neonatal health status during the short period of birth hospitalization. However, there was a tendency for a higher use of phototherapy in infants born with vitamin D deficiency as compared to those with vitamin D sufficiency (5.3 vs. 1.1%, one-sided *P* = 0.053). Generally, phototherapy is required in 1.4% of healthy born neonates [33]. Vitamin D deficiency has been reported in 86% infants with pathological hyperbilirubinemia, and 43% of controls [34]. To the best of our knowledge, no prior study has investigated the clinical status of neonates in association with CB vitamin D levels.

We acknowledge the potential limitations of our study. Maternal data that may correlate with neonatal vitamin D levels at birth, including the pre-pregnancy weight [35], skin color [13], and sun exposure during pregnancy [36] were not collected. However, it is unlikely that these limitations will affect the observed rate of vitamin D deficiency and insufficiency seen at birth in the studied infants. We did not find a difference between the maternal weight at delivery and occurrence of vitamin D deficiency/insufficiency in infants at birth. Moreover, a review of the literature revealed that data obtained from questionnaires regarding the assessment of sunlight exposure do not precisely estimate vitamin D status [37].

We would like to highlight the strengths of the present report that include: (i) prospective observation of a sufficient number and diverse population of mother-infant pairs, (ii) use of 95% Confidence Intervals to present the potential variability in prevalence of vitamin D deficiency/ insufficiency in CB, (iii) use of regression models for identifying maternal factors that increase the risk for vitamin D deficiency/insufficiency in infant at birth, and (iv) reporting clinical characteristics of the neonates in association with their vitamin D status at birth.

## Conclusions

A significant number of infants, especially those born to mothers of non-White race/ethnicity, younger age, and with a history of multiple pregnancies, are at risk for having insufficient and deficient levels of vitamin D in CB. Our data should encourage pediatricians to supplement infants with vitamin D especially considering the reported poor compliance with the AAP’s recommendations by both, pediatricians and parents [38]. Moreover, due to the high likelihood of vitamin D insufficiency/deficiency at birth, further studies may be required to determine the role of implementing vitamin D supplementation with respect to the CB vitamin D status.

## Abbreviations

25(OH) D_3_: Vitamin D_3_
25(OH)D: Total 25-hydroxyvitamin D
AAP: American Academy of Pediatrics
CB: Cord blood
EDTA: Ethylenediaminetetraacetic acid
LC/MS/MS: Liquid Chromatography/Tandem Mass Spectrometry

## References

[1] Dawodu A, Wagner CL (2012). Prevention of vitamin D deficiency in mothers and infants worldwide - a paradigm shift. Paediatr Int Child Health.

[2] Holick MF (2004). Sunlight and vitamin D for bone health and prevention of autoimmune diseases, cancers, and cardiovascular disease [review]. Am J Clin Nutr.

[3] Mylott BM, Kump T, Bolton ML, Greenbaum LA (2004). Rickets in the Dairy State. WMJ.

[4] Belderbos ME, Houben ML, Wilbrink B, Lentjes E, Bloemen EM, Kimpen JL, Rovers M, Bont L (2011). Cord blood vitamin D deficiency is associated with respiratory syncytial virus bronchiolitis. Pediatrics.

[5] Baïz N, Dargent-Molina P, Wark JD, Souberbielle JC, Annesi-Maesano I, EDEN Mother-Child Cohort Study Group (2014). Cord serum 25-hydroxyvitamin D and risk of early childhood transient wheezing and atopic dermatitis. J Allergy Clin Immunol.

[6] Hypponen E, Laara E, Jarvelin MR, Virtanen SM (2001). Intake of vitamin D and risk of type 1 diabetes: a birth-cohort study. Lancet.

[7] Hossein-nezhad A, Holick MF (2013). Vitamin D for health: a global perspective. Mayo Clin Proc.

[8] Wagner CL, Greer FR (2008). Prevention of rickets and vitamin D deficiency in infants, children, and adolescents. Pediatrics.

[9] Misra M, Pacaud D, Petryk A, Collett-Solberg PF, Kappy M (2008). Vitamin D deficiency in children and its management: review of current knowledge and recommendations. Pediatrics.

[10] Holick MF, Binkley NC, Bischoff-Ferrari HA, Gordon CM, Hanley DA, Heaney RP, Murad MH, Weaver CM (2011). Evaluation, treatment, and prevention of vitamin D deficiency: an endocrine society clinical practice guideline. J Clin Endocrinol Metab.

[11] Eldjerou LK, Cogle CR, Rosenau EH, Lu X, Bennett CA, Sugrue MW, Hoyne J, Lambert A, Ashley L, Sazama K, Fields G, Wingard JR, Zubair AC (2015). Vitamin D effect on umbilical cord blood characteristics: a comparison between African Americans and Caucasians. Transfusion.

[12] Jacquemyn Y, Ajaji M, Karepouan N (2013). Vitamin D levels in maternal serum and umbilical cord blood in a multi-ethnic population in Antwerp, Belgium. Facts Views Vis Obgyn.

[13] Cadario F, Savastio S, Pozzi E, Capelli A, Dondi E, Gatto M, Zaffaroni M, Bona G (2013). Vitamin D status in cord blood and newborns: ethnic differences. Ital J Pediatr.

[14] Bodnar LM, Simhan HN, Powers RW (2007). High prevalence of vitamin D insufficiency in black and white pregnant women residing in the northern United States and their neonates. J Nutr.

[15] Vinkhuyzen AA, Eyles DW, Burne TH, Blanken LM, Kruithof CJ, Verhulst F, Jaddoe VW, Tiemeier H, McGrath JJ (2015). Prevalence and predictors of vitamin D deficiency based on maternal mid-gestation and neonatal cord bloods: The Generation R Study. J Steroid Biochem Mol Biol.

[16] Bowyer L, Catling-Paull C, Diamond T (2009). Vitamin D, PTH and calcium levels in pregnant women and their neonates. Clin Endocrinol.

[17] Halm BM, Lai JF, Pagano I, Cooney W, Soon RA, Franke AA (2013). Vitamin D deficiency in cord plasma from multiethnic subjects living in the tropics. J Am Coll Nutr.

[18] Lee JM, Smith JR, Philipp BL (2007). Vitamin D deficiency in a healthy group of mothers and newborn infants. Clin Pediatr (Phila).

[19] http://www.questdiagnostics.com/testcenter/TestDetail.action?ntc=91935. Accessed 9 Oct 2015.

[20] Bailey D, Perumal N, Yazdanpanah M, Al Mahmud A, Baqui AH, Adeli K, Roth DE (2014). Maternal-fetal-infant dynamics of the C3-epimer of 25-hydroxyvitamin D. Clin Biochem.

[21] http://statisticalhorizons.com/multicollinearity. Accessed 30 Nov 2015.

[22] Chapuy MC, Preziosi P, Maamer M (1997). Prevalence of vitamin D insufficiency in an adult normal population. Osteoporos Int.

[23] Heaney RP, Dowell MS, Hale CA (2003). Calcium absorption varies within the reference range for serum 25-hydroxyvitamin D. J Am Coll Nutr.

[24] Holick MF (2009). Vitamin D, status: measurement, interpretation, and clinical application. Ann Epidemiol.

[25] Clemens TL, Adams JS, Henderson SL, Holick MF (1982). Increased skin pigment reduces the capacity of skin to synthesise vitamin D3. Lancet.

[26] Wolpowitz D, Gilchrest BA (2006). The vitamin D questions: how much do you need and how should you get it?. J Am Acad Dermatol.

[27] Mostafa WZ, Hegazy RA (2015). Vitamin D and the skin: Focus on a complex relationship: a review. Journal of Advanced Research.

[28] Sulaiman RA, Sharratt CL, Lee PW, Skinner A, Griffiths MJ, Webster C, Ford C, Anderson J, Gama R (2010). Ethnic differences in umbilical cord blood vitamin D and parathyroid hormone–South Asians compared to whites born in the UK. J Matern Fetal Neonatal Med.

[29] Andersen LB, Abrahamsen B, Dalgård C, Kyhl HB, Beck-Nielsen SS, Frost-Nielsen M, Jørgensen JS, Barington T, Christesen HT (2013). Parity and tanned white skin as novel predictors of vitamin D status in early pregnancy: a population-based cohort study. Clin Endocrinol (Oxf).

[30] Vercruyssen J, Martin M, Jacquemyn Y (2010). A pilot study on 25-hydroxyvitamin D status according to sun exposure in pregnant women in Antwerp, Belgium. Facts Views Vis Obgyn.

[31] Wegienka G, Kaur H, Sangha R, Cassidy-Bushrow AE (2016). Maternal-cord blood vitamin D correlations vary by maternal levels. J Pregnancy.

[32] Skouroliakou M, Ntountaniotis D, Massara P, Koutri K (2016). Investigation of multiple factors which may contribute to vitamin D levels of bedridden pregnant women and their preterm neonates. J Matern Fetal Neonatal Med.

[33] Atkinson LR, Escobar GJ, Takayama JI, Newman TB (2003). Phototherapy use in jaundiced newborns in a large managed care organization: Do clinicians adhere to the guideline?. Pediatrics.

[34] Mutlu M, Çayir A, Çayir Y, Özkan B, Aslan Y (2013). Vitamin D and hyperbilirubinaemia in neonates. HK J Paediatr (New Series).

[35] Bodnar LM, Catov JM, Roberts JM, Simhan HN (2007). Prepregnancy obesity predicts poor vitamin D status in mothers and their neonates. J Nutr.

[36] Ortigosa Gómez S, García-Algar O, Mur Sierra A, Ferrer Costa R, Carrascosa Lezcano A, Yeste FD (2015). Sociodemographic factors related to plasma concentrations of 25-OH vitamin D and PTH in cord blood. Rev Esp Salud Publica.

[37] McCarty CA (2008). Sunlight exposure assessment: can we accurately assess vitamin D exposure from sunlight questionnaires?. Am J Clin Nutr.

[38] Taylor JA, Geyer LJ, Feldman KW (2010). Pediatrics. Use of supplemental vitamin d among infants breastfed for prolonged periods. Pediatrics.

